# AbAdapt: an adaptive approach to predicting antibody–antigen complex structures from sequence

**DOI:** 10.1093/bioadv/vbac015

**Published:** 2022-03-07

**Authors:** Ana Davila, Zichang Xu, Songling Li, John Rozewicki, Jan Wilamowski, Sergei Kotelnikov, Dima Kozakov, Shunsuke Teraguchi, Daron M Standley

**Affiliations:** 1 Research Institute for Microbial Diseases, Department of Genome Informatics, Osaka University, Suita 565-0871, Japan; 2 Department of Applied Mathematics and Statistics, Stony Brook University, Stony Brook, NY 11794-5252, USA; 3 Laufer Center for Physical and Quantitative Biology, Stony Brook University, Stony Brook, NY 11794-5252, USA; 4 Faculty of Data Science, Shiga University, Hikone 522-8522, Japan; 5 Immunology Frontier Research Center, Department of Systems Immunology, Osaka University, Suita 565-0871, Japan

## Abstract

**Motivation:**

The scoring of antibody–antigen docked poses starting from unbound homology models has not been systematically optimized for a large and diverse set of input sequences.

**Results:**

To address this need, we have developed AbAdapt, a webserver that accepts antibody and antigen sequences, models their 3D structures, predicts epitope and paratope, and then docks the modeled structures using two established docking engines (Piper and Hex). Each of the key steps has been optimized by developing and training new machine-learning models. The sequences from a diverse set of 622 antibody–antigen pairs with known structure were used as inputs for leave-one-out cross-validation. The final set of cluster representatives included at least one ‘Adequate’ pose for 550/622 (88.4%) of the queries. The median (interquartile range) ranks of these ‘Adequate’ poses were 22 (5–77). Similar results were obtained on a holdout set of 100 unrelated antibody–antigen pairs. When epitopes were repredicted using docking-derived features for specific antibodies, the median ROC AUC increased from 0.679 to 0.720 in cross-validation and from 0.694 to 0.730 in the holdout set.

**Availability and implementation:**

AbAdapt and related data are available at https://sysimm.org/abadapt/.

**Supplementary information:**

Supplementary data are available at *Bioinformatics Advances* online.

## 1 Introduction

One of the hallmarks of the adaptive immune system is its ability to quickly mount a defense against any ‘non-self’ molecule (antigen). A robust immune response requires continuous generation of a vast repertoire of lymphocyte receptors. Among these, the B-cell receptors (BCRs), and their soluble form, antibodies, play a critical role in host defense. BCRs are initially generated from randomly selected V, (D) and J germline gene segments that code for one of two polypeptide chains (heavy or light). The vast majority of such candidate BCR sequences are eliminated as a result of binding to endogenous proteins. Those that survive and recognize nonself antigens can further develop by a process of rapid mutation and affinity-driven selection known as *maturation*. In this way, mature antibodies acquire binding sites (paratopes) that are optimized, both in terms of sequence and structure, to recognize specific antigen-binding sites (epitopes). Recent single-cell resolution sequencing technologies, which allow large numbers of paired (heavy–light) antibody sequences to be obtained in a single experiment, make it possible to quickly sample antibody repertoires from disease cohorts. In the recent COVID-19 pandemic, for example, a number of researchers reported human antibody sequences against the SARS-CoV-2 S protein within months of the outbreak (Brouwer *et al.*, 2020; Cao *et al.*, 2020; Chi *et al.*, 2020).

In order to functionally characterize such antibodies, we generally need to know their binding modes. Traditionally, experimental methods such as X-ray crystallography have been used for this purpose. However, because these experimental methods are low throughput, there are far more antibody sequences of interest than there are antibody–antigen complex structures. There is, therefore, a need for computational methods that can leverage existing structural data to predict novel antibody–antigen complexes from emerging antibody sequence data.

**Fig. 1.**
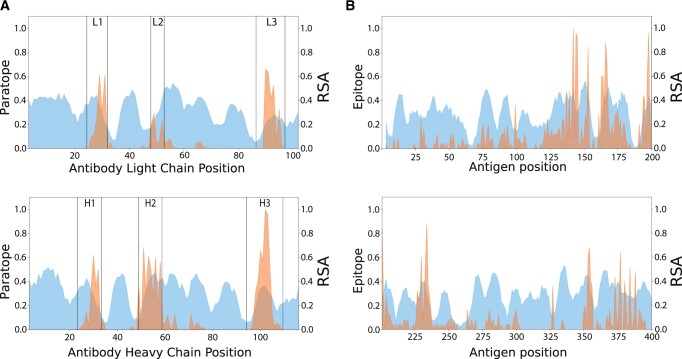
Comparison between paratopes and epitopes. (**A**) The observed paratopes in known antibody–antigen complexes are indicated by a histogram of contacts (orange). The CDR boundaries are indicated by vertical gray lines. The light blue areas indicate RSA. (**B**) Influenza hemagglutinin epitopes in known antibody–hemagglutinin complexes are indicated in orange, while light blue areas indicate RSA

To a first approximation, the prediction of antibody–antigen complex structures can be separated into two problems: paratope prediction and epitope prediction. Of the two, epitope prediction is the more difficult. This can be understood by examining the frequency of paratope residues in all known antibody–antigen complexes and comparing this distribution to that of the known epitope residues for even a single antigen. Virtually all known paratopes lie within or near the three ‘complementarity-determining regions’ (CDRs), as shown in Figure 1A. In contrast, as illustrated using Influenza hemagglutinin as a typical antigen, epitopes can be comprised of almost any surface-exposed residue (Fig. 1B). It is for this reason that the epitope prediction problem is considered incomplete without specification of the antibody of interest (Sela-Culang *et al.*, 2015). Many epitope prediction methods have been proposed; however, most do not make use of antibody information.

One natural way of making use of antibody information in epitope prediction is through molecular docking. Numerous docking engines exist, of which several—ClusPro (Kozakov *et al.*, 2017), PatchDock (Schneidman-Duhovny *et al.*, 2005), FRODOCK (Ramírez-Aportela *et al.*, 2016) and SnugDock (Sircar and Gray, 2010)—provide antibody-specific modes in which CDRs are masked and, in the case of ClusPro, antibody-specific scoring functions are employed. Docking-derived features can then be used in the prediction of antibody-specific epitopes. An early example of work in this direction is EpiPred, which utilized docking scores and geometric constraints to infer epitopes (Krawczyk *et al.*, 2014). In related works, Jespersen *et al.* (2019) developed a machine-learning method to distinguish true antibody–antigen interfaces from false ones, and Pittala and Bailey-Kellogg (2020) used an attention-based deep-learning framework to capture the context of the target protein, paratope or epitope.

In a complementary study, Ambrosetti *et al.* (2020a) demonstrated that a rough approximation of the epitope significantly improved docking accuracy using the program HADDOCK (Dominguez *et al.*, 2003). Since docking can assist in epitope/paratope prediction and vice versa, it is natural to try to integrate these two approaches along with antigen/antibody 3D modeling. However, while such integration is conceptually straightforward, docking methods are typically very sensitive to structural modeling errors in practice (Anishchenko *et al.*, 2017). Because paratopes tend to occur in or near CDRs, which are precisely the most difficult regions in antibodies to model accurately (Almagro *et al.*, 2014), automated antibody modeling and docking from sequence remains a challenging problem.

**Fig. 2.**
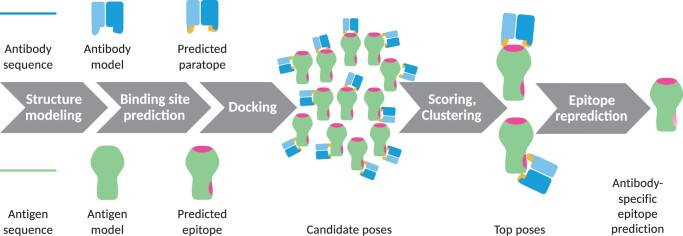
Flowchart of AbAdapt pipeline. The pipeline transforms an antibody–antigen sequence pair in order to produce a set of top-scoring poses, which are then used to repredict the epitope

Here, we describe a webserver, AbAdapt (https://sysimm.org/abadapt/), that employs established methods for antigen/antibody modeling and docking, and couples these methods using machine learning at key decision points. The basic workflow used by AbAdapt is illustrated in Figure 2. The key points are as follows: Automated antibody and antigen structural modeling pipelines have been built in order to allow direct input of sequences. Methods for epitope and paratope prediction were developed and trained using sequence and structural features derived from the antibody and antigen models. Two docking engines—Piper (Kozakov *et al.*, 2006) and Hex (Macindoe *et al.*, 2010)—were employed in order to perform both global and local docking, respectively, consistent with epitope predictions. Scoring functions were developed for raw Piper poses, raw Hex poses, and also for coclustered Piper and Hex poses. Epitope residues were finally re-predicted based on top-scoring docked poses. To test AbAdapt, we attempted to simulate a situation where the antibody and antigen sequence were known, but the epitope was not. For the antibodies, we employed template blacklisting in the structural modeling step in order to introduce realistic noise expected when modeling new antibody sequences. For the antigen, we only blacklisted templates that shared an epitope with the query, as would be the case for most well-studied antigens (e.g. Influenza hemagglutinin or SARS-CoV-2 spike protein), for which the unbound structure is known. We performed leave-one-out cross-validation (LOOCV) using 622 nonredundant antibody–antigen sequence pairs with known structure. We further evaluated the performance on an independent holdout set comprised of 100 randomly chosen, sequence-unique antibody–antigen pairs.

## 2 Methods

### 2.1 Datasets

The datasets for training and testing were prepared from Protein Data Bank (PDB) entries (Berman, 2000) containing crystal structures of antibody–antigen complexes (downloaded May 2021). Redundant complexes were filtered according to the following criteria: Resolution better than 4 Å and complete heavy and light variable chains and antigen length greater than or equal to 60 amino acids. This filtering resulted in 1862 Ab–Ag complexes, which were further filtered, as described below.

### 2.2 Antibody and antigen 3D models

Antibody variable regions were modeled using Repertoire Builder (Schritt *et al.*, 2019). In order to introduce a realistic level of noise, templates with a sequence identity of 90% or higher were blacklisted from the modeling procedure. Antigen templates were restricted to PDB entries that either did not have a bound antibody or whose bound antibody overlapped <50% with the residues of the native epitope. Template alignments were computed by Forte (Tomii and Akiyama, 2004), rendered in 3D using Spanner (Lis, 2011) followed by side chain replacement by SCWRL4 (Krivov *et al.*, 2009). We excluded 290 Ab–Ag pairs because the Root Mean Square Deviation (RMSD) of either the paratope or epitope were higher than 6 Å.

### 2.3 Redundancy filtering

The antibody–antigen pairs were redundancy filtered as follows: a pseudosequence composed of antibody heavy and light chain variable domains was constructed; the sequences were clustered using CD-HIT (Fu *et al.*, 2012) at an 85% identity threshold. Antigens that were themselves antibodies (5) or that caused any of the third-party software to fail (1) were removed. This filtering resulted in 722 nonredundant antibody–antigen model pairs.

### 2.4 Paratope and epitope prediction

In order to generate input for the paratope prediction model, antibody CDR sequences were annotated with the AHo numbering scheme (Honegger and Plückthun, 2001) using the ANARCI program (Dunbar and Deane, 2016), ±2 flanking amino acid residues to capture potential non-CDR residues involved in the binding. For training, a binding residue in the antigen (antibody) was defined as True if it contained an atom that was within 5 Å of any antibody (antigen) atom. The paratope feature vector consisted of three sequence-based terms (amino acid, physico-chemical properties and conservation) and two structure-based terms [accessible surface area (ASA) and surface shape]. The epitope feature vector consisted of only the amino acid, conservation, ASA and surface shape terms. The feature vector of each residue of interest was concatenated over a window of ±5 amino acids.

The individual features were defined as follows: The residue types for 20 standard amino acids were defined using one-hot encoding; A histogram of nine states (Tiny, Small, Aliphatic, Aromatic, Nonpolar, Polar, Charged, Basic and Acidic), along with the overall isoelectric point for the sequence window of interest, as defined by the R package SeqinR (Charif and Lobry, 2007); A position-specific scoring matrix based on HHBlits (version 2.0.15) as calculated by SPBuild (Yamada and Kinoshita, 2018); the ASA for each amino acid residue, as computed using DMS (Richards, 1977); deformation of the protein surface using the Laplacian Norm (LN), a measure of curvature at five levels of resolution (Bonnel and Marteau, 2012), as described in Li *et al.* (2014).

The ML models for paratope (*Initial_Paratope_ML*) and initial epitope (*Initial_Epitope_ML*) are summarized in Supplementary Tables S1 and S2. For paratope and epitope prediction, a deep neural network (DNN) was designed with fully connected (dense) layers using the TensorFlow (Abadi *et al.*, 2016) python API. The input layer received the feature vector. Subsequent 7 (11) hidden layers for the paratope (epitope) predictor transformed the inputs nonlinearly using Rectified Linear Unit (ReLU) activation functions. We adopted batch normalization (Ioffe and Szegedy, 2015) right after each hidden layer and before the activation function, starting from the second hidden layer. The network ends with a sigmoid activation function to output epitope or paratope probabilities. The DNN performed binary classification (0, 1) representing nonbinding or binding for each residue of interest. The DNN was trained with the binary cross-entropy loss function and optimized using Adam, a stochastic gradient-based minimizer for the loss function (Kingma and Ba, 2014). Overfitting was prevented by randomly dropping units from the hidden layers during training (Srivastava *et al.*, 2014; the dropout rates were tuned separately for each layer); and stopping training when the loss no longer decreased (Prechelt, 1998). The class-imbalance issue was addressed by setting the class weight equal to one-half of the ratio of the antigen length to the residues in the class (binding or nonbinding). Hyperparameter tuning and model selection were performed using the RandomSearch algorithm (Bergstra and Bengio, 2012) from the Keras Tuner library; https://github.com/keras-team/keras-tuner/) through 10-fold nested cross-validation. Unless otherwise specified, precision and Recall were evaluated at a binary cutoff of 0.5.

### 2.5 Docking

Two established docking engines, Piper and Hex, were used in this study. Piper, the docking engine used by ClusPro, has consistently ranked at the top in the CAPRI (Critical Assessment of PRedicted Interactions) docking assessment and utilizes Fast Fourier Transformation (FFT) in Cartesian coordinate space in order to globally sample translations (Kozakov *et al.*, 2006). Piper version 1.2 was run using default settings for global antibody–antigen docking. All 70 000 poses were evaluated by converting the transformations to Cartesian coordinates and scoring, as described below.

Hex (Macindoe *et al.*, 2010), which utilizes FFT in spherical coordinates, was used for local antibody–antigen docking at specific epitope patches. First, residues were sorted by decreasing epitope probability and subsequently clustered into nonidentical patches of radius 20 Å. The patch center was set to the residue with the highest epitope probability, with distance between patch centers at least 10 Å. Only patches with summed probability at least half the value of the highest-scoring patch were retained. Each patch was used for an independent Hex antibody–antigen docking calculation centered on the epitope patch of interest, with antibody–antigen orientations constrained to ±30° from their intermolecular axis.

Piper and Hex Poses were scored by the *Piper_Docking_ML* or the *Hex_Docking_ML*, respectively, as summarized in Supplementary Table S1. Each Hex or Piper pose was described by a rotationally invariant, compact feature vector containing the following terms: the ratio of observed to expected contacts for each of the 20 standard amino acids (assumed to be proportional to the total relative surface area; Hubbard and Thornton, 1993) on the antibody; the analogous 20 ratios for the antigen; number of interatomic clashes; the joint-frequencies contacts/noncontacts in agreement/disagreement with paratope and epitope predictions.

With regard to the joint frequency features, we considered antigen and antibody residue contacts separately and analogously. Using the antigen as an example, we first defined the joint frequency of two variables: observed (contact and noncontact) and predicted (epitope and nonepitope), where a binary cutoff of 0.5 was used for both paratope and epitope. These frequencies were normalized by the total number of residue contacts. We then discarded the large noncontact/nonepitope term and retained the three others. Together, the joint frequency features thus consisted of three antigen and three antibody terms.

Poses were labeled as ‘True’ or ‘False’ by comparing them to an ideal pose that had been built by superimposing the antibody and antigen models on the native using paratope and epitope residues, respectively. True poses had to meet three criteria: the backbone RMSD of the interface residues (IRMSD), as defined by Kozakov *et al.* (2017), had to be less than a threshold (*rmsdTrue*); the fraction of antibody contacts that were true had to be greater than a threshold (*parTrue*); and, the fraction of antigen contacts that were true had to be greater than a threshold (*epTrue*). If one or more of these criteria were not met, the pose was deemed False.

The docking score was trained using the XGBoost Python module XGBClassifier with true features scaled by the ratio of the numbers of True to False poses. Poses were randomly downsampled to 10 000 in training.

### 2.6 Coclustering and rescoring Piper and Hex poses

The top 10 000 poses, 10 000×fPiper from Piper and 10 000×(1−fPiper) from Hex, were selected according to the scores described above. In order to assess the poses on a common scale, they were coclustered progressively by IRMSD. Cluster representatives were defined as the member with the most neighbors within the IRMSD clustering cutoff (10 Å). For each cluster representative, a feature vector consisting of the following terms was constructed: AbAdapt Hex or Piper score; number of interatomic clashes; Hex or Piper raw energy; fraction of Piper poses in the cluster; total size of cluster. The final docking score (*Piper_Hex_Docking_ML*) was trained using the XGBoost Python module XGBRegressor, with the target function given by: $\frac{1}{\mathrm{IRMS}D^{2}}$. We assessed the quality of the top-scoring poses by first labeling each cluster representative as True or False, sorting the poses by their final score, and, if a True poses existed, computing the rank of the top-ranked True cluster representative (True Rank). In this scheme, a True Rank of 1 would be perfect. A failure would be when no True pose exists among cluster representatives. An intermediate result would be when a True cluster representative exists, but has a rank greater than 1. The upper limit on the rank in such a case would be defined by the number of clusters.

### 2.7 Antibody-specific epitope prediction

In order to include information from antibody–antigen docking in the final epitope prediction, a new feature vector was constructed that made use of the conservation, ASA and surface shape terms along with additional docking-derived features. The additional features describe the distribution of combined Piper–Hex scores associated with a given antigen residue. Specifically, we computed the joint frequency *f*(*i*, *s*) of a given antigen residue *i* being in contact with the antibody and having a *Piper_Hex_Docking_ML* score *s*. This joint frequency feature should not be confused with that used in Section 2.5, in which the initial epitope and paratope predictions were used; rather the epitope and paratope in *f*(*i*, *s*) come from the structure of the pose. Here, *s* was discretized into one of 10 equally spaced bins. The final predictor model (*Final_Epitope_ML*) was a DNN designed with seven fully connected (dense) hidden layers using the ReLU activation function, and sigmoid activation function for the output layer. The network used the Adam optimizer (Kingma and Ba, 2014), and regularization methods batch normalization, Dropout (Srivastava *et al.*, 2014) and early stopping. Supplementary Table S2 summarizes the architecture.

### 2.8 Restraints

In order to allow the incorporation of additional information on the binding site, epitope restraints (not used in the present study) were implemented as follows. First, the epitope probability for any antigen residue can be set manually, and this probability will override the initial epitope prediction. Second, a global parameter (*rrest*) can be provided such that the epitope probability of any residue whose C-alpha atom is more than *rrest* from any restrained epitope residue will be set to zero. The epitope probabilities of residues not outside this distance and not manually restrained will be determined by the initial epitope prediction (*Initial_Epitope_ML*), as above. The restraints thus work purely at the level of the initial epitope prediction, and act to remove docked poses that are far from restrained residues. There is no additional restraint term in the final scoring function. If the binding site is actually known with a high degree of confidence, we recommend using a data-driven docking method like HADDOCK (Dominguez *et al.*, 2003).

## 3 Results

### 3.1 Antibody and antigen modeling

Separate structural models for antibody and antigen sequences were constructed, as described in Section 2. In brief, we used blacklisting of antibody templates with 90% or more sequence identity to the query sequences, and excluded any antigen templates with a bound antibody sharing the same epitope. In order to simplify the downstream pose assessment, a reference complex was constructed by superimposing the paratope and epitope residues of these models onto those of the native structure. We used the reference, rather than the actual native, in all RMSD calculations of docked poses. Generally, RMSDs to the reference will be lower than to the actual native, and a pose can have zero RMSD even if the antibody model is imperfect. The mean and median C-alpha RMSDs of paratope residues upon superposition were 2.71 Å and 2.46 Å, while those of the epitope residues were 1.18 Å and 0.794 Å, respectively. The fact that epitope RMSDs were generally lower than paratope RMSDs was a consequence of the modeling procedure, which did not disallow sequence-identical antigen templates, so long as they were not solved with an antibody bound to the epitope in question.

### 3.2 Abadapt leave-one-out cross-validation

LOOCV was performed on the entire AbAdapt pipeline using the 622 separate antibody and antigen models. That is, for each machine-learning step, 622 models were constructed for each query using the remaining 621 queries as training data. The actual docking calculations were performed only once, as pose generation was independent of scoring. Several global parameters (defined in Section 2) were fixed in the results below: *rmsdTrue* = 15 Å; *epTrue*= *parTrue* = 0.5; *fPiper* = 0.9. In the following, we break down the performance of each of the main steps of the AbAdapt pipeline in LOOCV.

#### 3.2.1 Initial paratope prediction

**Fig. 3.**
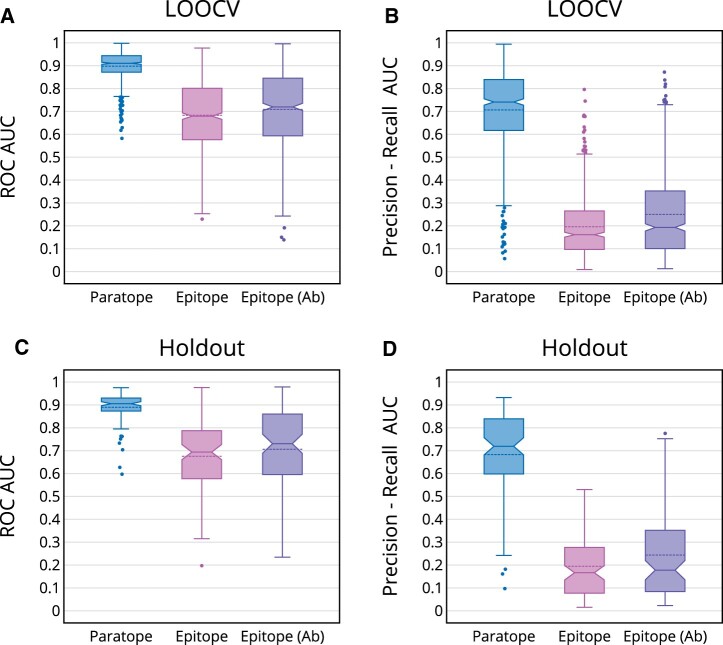
Paratope and initial epitope predictions in LOOCV and holdout. The ROC (**A**, **C**) and PR (**B**, **D**) AUCs are shown for paratope, epitope and antigen-specific epitope predictions

The Receiver Operating Characteristic (ROC) Area Under the Curve (AUC) and Precision-Recall (PR) AUC for the *Initial_Paratope_ML* in LOOCV were assessed (Fig. 3A and B; Table 1). The mean ROC AUCs for the training (92.5%) and test (89.8%) were close, consistent with the observation that paratopes tend to be located in predictable regions.

**Table 1.** LOOCV initial paratope and epitope predictions

Prediction	Median	Mean	Stdev
Paratope TRAIN ROC AUC	0.925	0.925	0.011
Paratope TEST ROC AUC	0.910	0.898	0.064
Paratope TEST PR AUC	0.741	0.707	0.184
Paratope TEST recall	0.816	0.797	0.135
Paratope TEST precision	0.621	0.591	0.178
Paratope TEST positive rate	0.239	0.232	0.066
Epitope TRAIN ROC AUC	0.861	0.853	0.050
Epitope TEST ROC AUC	0.679	0.683	0.147
Epitope TEST PR AUC	0.161	0.196	0.134
Epitope TEST recall	0.625	0.599	0.240
Epitope TEST precision	0.146	0.156	0.087
Epitope TEST positive rate	0.086	0.100	0.069

#### 3.2.2 Initial epitope prediction

The ROC and PR AUCs for the *Initial_Epitope_ML* in LOOCV were computed (Fig. 3A and B; Table 1). All measures were lower than their corresponding values in paratope prediction, consistent with the inherent limitation in predicting epitope residues without specification of the antibody. In particular, the mean PR AUC for epitope prediction (19.6%) was much lower than in the paratope prediction (70.7%). Though this is partially explained by the difference of the positive rates, which gives the baseline of the PR AUC, the reduction is much more severe than what is expected from the positive rate alone. This highlights the fundamental difficulty of epitope prediction. We note also that the gap between mean test and training ROC AUCs was significant (*P* < 0.0001, one-tailed *t*-test), implying that the current epitope predictor does not generalize well to unseen data. In spite of the fundamental difficulty of epitope prediction using only antigen features, the performance of AbAdapt’s initial epitope prediction dropped further if a simplified feature vector, consisting only of the antigen relative solvent accessibility (RSA) of each residue, was used. Here, the mean ROC AUCs dropped from 0.683 to 0.637 and mean PR AUC dropped from 0.196 to 0.142. We concluded that a natural direction to address this difficult problem is to include antibody features in the epitope prediction model, an idea that we explore below.

#### 3.2.3 Piper docking

**Fig. 4.**
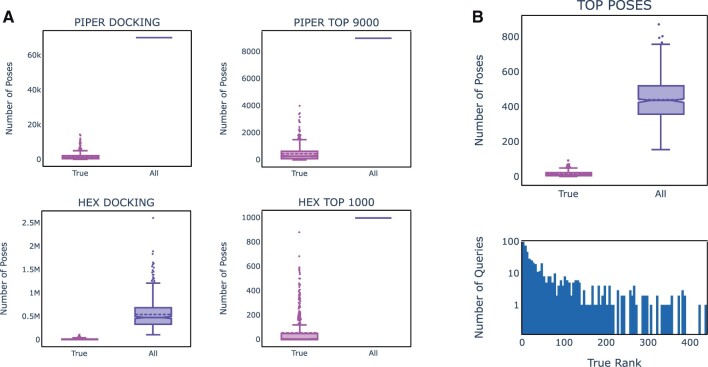
Distribution of True poses in LOOCV. The distribution of True and All poses are shown for five stages of the AbAdapt pipeline. (**A**) Piper raw docking (upper left) and selected poses (upper middle); Hex raw docking (lower left) and selected poses (lower middle); (**B**) Final cluster representatives (right). A histogram of True Ranks of cluster representative in the final set of poses is also shown (blue bars)

The number of Piper runs that produced at least one True pose out of the 70 000 poses generated per query was 596/622 (95.8%). The mean ROC AUCs for the *Piper_Docking_ML* for these 596 queries was 0.692 (±0.151). When raw piper energies were used instead of *Piper_Docking_ML*, the mean ROC AUC dropped to 0.538 (±0.147). The AbAdapt features thus had a significant effect on the scoring. Nevertheless, the proportion of True poses, as illustrated in Figure 4A, made up only a tiny proportion (2.5% on average) of the Piper poses initially. We defined the True Rank as the rank of the highest-scoring pose that is classified as True. The median [interquartile range (IQR)] True Rank was 5988 (2423–12 931). Below, we attempt to reduce the True Rank by merging with Hex poses, clustering and rescoring.

#### 3.2.4 Hex docking

The number of Hex runs that produced at least one True pose out of all poses was 583/622 (93.7%). The total number of poses depended on the number of high-scoring epitope patches, with median (IQR) values of 472 087 (327 281–681 936; Fig. 4B). The mean ROC AUCs for the *Hex_Docking_ML* for the 583 queries that produced at least one True pose was 0.599 (±0.156). When we removed epitope and paratope patch information from the Hex docking protocol, the query coverage dropped to 104/622 (16.7%), indicating the need for such patch-directed local docking in the case of Hex. When we utilized the AbAdapt Hex docking protocol but used raw Hex scores, the mean ROC AUC dropped to 0.528 (±0.085). Taken together, both AbAdapt sampling and scoring had a positive effect on overall Hex docking performance. Nevertheless, as observed with Piper, True poses made up a tiny proportion (3.1% on average) of the Hex poses initially. The median (IQR) True Rank was 5639 (1208–25 757), which, again, we aimed to reduce by merging with Piper poses, clustering and rescoring.

#### 3.2.5 Combined Piper–Hex clustering and scoring

The top Piper and Hex poses were next coclustered and rescored. As illustrated in Figure 4A, the first step involved selection of Piper and Hex poses. In the first selection of 9000 from 70 000 Piper poses, the mean True pose frequency increased from 2.4% to 4.8%. For Hex, the True poses frequency increased from 3.1% to 5.4% among the top 1000 selected poses. The second step involved rescoring the mixed Piper and Hex poses using regression against a target function given by $\frac{1}{\mathrm{IRMS}D^{2}}$, and then clustering the poses (Fig. 4B). Here, we actually observed a drop in the enrichment of True poses to 3.9% for cluster representatives. The *Piper_Hex_Docking_ML* was assessed for cluster representatives by computing the overall coverage and True Rank for each query. The overall query coverage of cluster representatives, was 550/622 (88.4%). The True Rank histogram (Fig. 4B), had a very long tail, with a median (IQR) value of 22 (5–77). Thus, the loss in overall True pose enrichment was compensated by an overall improvement in True Rank. The mean ROC AUC for the combined *Piper_Hex_Docking_ML* was 0.533 (±0.156). This value was lower than that obtained if we had simply inherited the *Piper_Docking_ML* or *Hex_Docking_ML* scores (0.554 ± 0.198). However, the regression provided at least two benefits: first, it provided a single score for poses from either Piper or Hex; second, it allowed poses with IRMSD values well below the cutoff of 15 Å to influence the score, since lower IRMSD values are always preferred in $\frac{1}{\mathrm{IRMS}D^{2}}$ regression. For example, if we considered a stricter IRMSD cutoff of 8 Å, we found that the mean ROC AUC increased to 0.608 (±0.241) when the regression score was used but only 0.570 (±0.235) when *Piper_Docking_ML*/*Hex_Docking_ML* was used. The query coverage using the 8 Å cutoff was, as expect, lower: 368/622 (59%).

As a reference, we also constructed two alternative pipelines, in which only-Piper (*fPiper* = 1) or only-Hex (*fPiper* = 0) were used. For the only-Piper pipeline, the query coverage was 549/622 (88.26%) and the median (IQR) True pose ranks were 19 (6–70). For the only-Hex pipeline, the query coverage was 459/622 (73.79%) and the median (IQR) True pose ranks were 20 (6–71.5). From these results, including Hex did not appear to have obvious advantages over the only-Piper pipeline in terms of query coverage or True Rank. However, if we again look below the 15 Å IRMSD threshold, we found that the query coverage of the Piper–Hex pipeline (368) was much better than that of the only-Piper pipeline (340). Therefore, the best value of (*fPiper*) depends on both the inputs and the desired output.

**Fig. 5.**
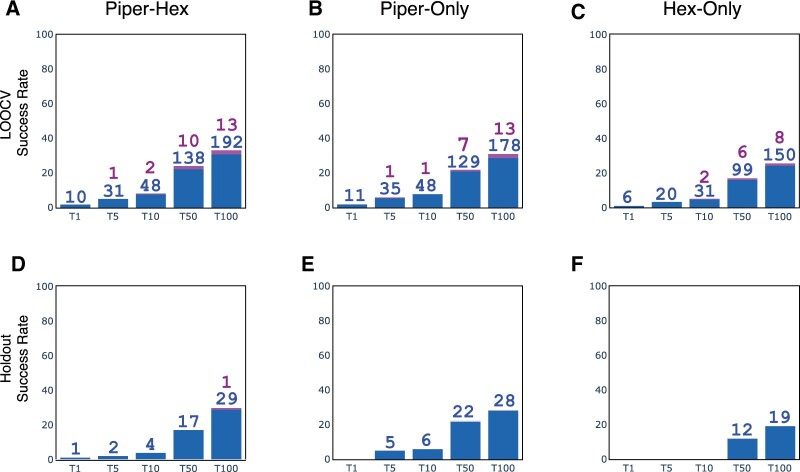
DockQ scores in LOOCV and holdout. (**A**, **D**) The total number of Acceptable, Medium and High models for the 622 (100) antibody–antigen pairs used in cross-validation (Holdout) are shown. (**B**, **C**) The corresponding values are shown for a reference pipeline in which only-Piper was used. (**C**, **E**) The corresponding values are shown for a reference pipeline in which only-Hex was used

In order to put these results into a more familiar context, we evaluated DockQ scores (Basu and Wallner, 2016), which map to the four categories (‘Incorrect’, ‘Acceptable’, ‘Medium’ or ‘High’) used in the CAPRI community (Lensink and Wodak, 2013). We summarized the DockQ results by plotting the frequency of queries resulting in at least one Acceptable, Medium or High model within the top 1, 5, 10, 50 or 100 cluster representatives (Fig. 5A). We also computed DockQ results for the two alternative pipelines in which only-Piper or only-Hex were used (Fig. 5B and C, respectively). For all three pipelines, no models deemed High were observed, and even Medium models were very rare, which indicates the limits of our current pipeline. Only two of the categories had frequencies above 10%: Acceptable models in the top 50 or top 100 poses, which is consistent with the True Ranks computed in Figure 4. In the top 50 poses, the only-Hex, only-Piper and combined Piper–Hex docking pipelines achieved query frequencies of 16%, 21% and 22%, respectively. Within the top 100 poses, the frequencies were 24.1%, 28.6% and 30.9%, respectively. Taken together, the combined pipeline appeared to out-perform the only-Hex or only-Piper pipelines in LOOCV.

One of the goals of this work was to develop a docking pipeline that was robust against errors in the initial models. Due to our use of blacklisting in the antibody modeling process, most of the errors in initial models came from the paratopes. We examined sensitivity of AbAdapt to antibody model quality in several ways. First, we considered the ability to generate at least one True pose (query coverage) for queries whose paratope RMSDs fell into three ranges: ‘Low’ 0–2 Å (201 cases), ‘Medium’ 2–4 Å (332 cases) and ‘High’ 4–6 Å (89 cases). *P-*values were calculated by Fisher’s exact test under the null hypothesis that the ability to produce at least one True pose in the final set of cluster representatives and paratope RMSD bin were independent of each other (Supplementary Table S3). We found that, while the ‘Low’ and ‘Medium’ success rates were not significantly different, the ‘High’ RMSD models failed significantly more than the ‘Low’ (*P* = 0.0025) or ‘Medium’ (*P* = 0.0039) models. Since our definition of a True pose was admittedly very liberal, we next considered the stricter definition of Acceptable according to the DockQ score for models within the top 10, 50 or 100 poses. Here again, we observed significant differences in success rates, depending on paratope RMSD, in particular between ‘Low’ and ‘High’ RMSD models in the top 100 poses (*P* = 0.00038). Taken together, AbAdapt was sensitive to model quality, consistent with previous reports (Kundrotas *et al.*, 2018).

### 3.3 Abadapt performance on holdout set

**Fig. 6.**
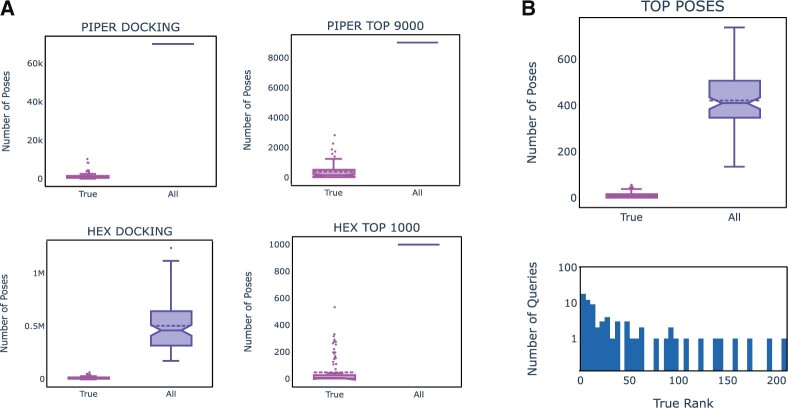
Distribution of True Poses in holdout set. The distribution of True and All poses are shown for five stages of the AbAdapt pipeline. (**A**) Piper raw docking (upper left) and selected poses (upper middle); Hex raw docking (lower left) and selected poses (lower middle); (**B**) Final cluster representatives (right). A histogram of True Ranks of cluster representative in the final set of poses is also shown (blue bars)

The 100 antibody–antigen holdout queries were modeled in an analogous manner to those of the cross-validation set. Here, all machine-learning models were trained on the entire 622 training cases. The paratope and epitope prediction performances are plotted in Figure 3C and D and summarized in Table 2. Overall, these values are very close to those in the cross-validation tests, indicating that the LOOCV results were a fair representation of how well the models generalized to new data. We summarized the main docking performance indicators in Table 3 and illustrated the distributions of True poses in Figure 6.

**Table 2.** Holdout initial paratope and epitope predictions

Prediction	Median	Mean	Stdev
Paratope TEST ROC AUC	0.905	0.890	0.066
Paratope TEST PR AUC	0.719	0.683	0.183
Paratope TEST recall	0.815	0.788	0.141
Paratope TEST precision	0.613	0.574	0.173
Paratope TEST positive rate	0.239	0.225	0.065
Epitope TEST ROC AUC	0.694	0.675	0.152
Epitope TEST PR AUC	0.166	0.194	0.134
Epitope TEST recall	0.625	0.572	0.252
Epitope TEST precision	0.151	0.151	0.090
Epitope TEST positive rate	0.086	0.093	0.054

**Table 3.** Comparison between cross-validation, holdout and ZDOCK

	LOOCV	Holdout	ZDOCK
Number of queries	622	100	43
Mean paratope RMSD (Å)	2.69	2.83	2.15
Median paratope RMSD (Å)	2.44	2.585	2.065
Mean epitope RMSD (Å)	1.20	1.03	1.021
Median epitope RMSD (Å)	0.795	0.732	0.726
Only-Piper query coverage	0.883	0.83	0.93
Only-Hex query coverage	0.738	0.72	0.93
Combined query coverage	0.884	0.83	0.93
Combined mean True Rank	61.3	66.3	29.6
Combined median true Rank	22	19	12
Combined mean ROC AUC	0.618	0.602	0.552
Combined median ROC AUC	0.554	0.537	0.544

As with the cross-validation runs, we assessed the final holdout poses using the DockQ score (Fig. 5). Overall, a similar trend was observed, in which both the combined Piper–Hex and Piper-only pipelines produced greater numbers of Acceptable models at each of the rank thresholds than the Hex-only pipeline.

### 3.4 Performance on ZDOCK benchmark

We next assessed AbAdapt’s performance on the subset of 43 antibody–antigen pairs in the ZDOCK Benchmark (Vreven *et al.*, 2015). The ZDOCK benchmark provides structures for the separate antibody and antigen, as well as for the bound complex. We assessed AbAdapt on the ZDOCK Benchmark under three conditions: (i) using bound structures; (ii) using unbound structures; and (iii) using models prepared in a manner identical to the way models were prepared for LOOCV and holdout runs. We summarize the results using models in Table 3. The AbAdapt coverage and True Rank were higher than for the LOOCV or holdout runs, although the quality of input structures was similar. We also computed DockQ results for the three conditions, as shown in Supplementary Figure S3. As expected, the numbers of high- and medium-quality models were greatest for the bound scenario, followed by the unbound PDB files, followed by the models.

### 3.5 Antibody-specific epitope predictions

In order to include antibody information in the epitope prediction, additional postdocking epitope features were derived from docked complexes. The additional features described the joint-frequencies of residue-level antibody–antigen contacts and docking scores. The addition of these postdocking features resulted in an improvement in median ROC AUC from 0.679 to 0.718 in LOOCV, as shown in the ‘Epitope (Ab)’ plot in Figure 3A and summarized in Table 4. When the postdocking features were applied to the holdout set, the median test ROC AUC for epitope prediction improved from 0.694 to 0.722, as shown in Figure 3C and summarized in Table 4.

**Table 4.** LOOCV and holdout for antibody-specific epitope predictions

Prediction	Median	Mean	Stdev
LOOCV TRAIN ROC AUC	0.796	0.798	0.018
LOOCV TEST ROC AUC	0.720	0.709	0.170
LOOCV TEST PR AUC	0.193	0.250	0.193
LOOCV TEST precision	0.154	0.161	0.098
LOOCV TEST recall	0.750	0.668	0.295
Holdout TEST ROC AUC	0.730	0.706	0.170
Holdout TEST PR AUC	0.178	0.244	0.185
Holdout TEST precision	0.155	0.158	0.087
Holdout TEST recall	0.726	0.656	0.268

In addition to the benchmarks above, we summarize the antibody-specific epitope prediction performance for AbAdapt along with EpiPred (Krawczyk *et al.*, 2014) and PECAN (Pittala and Bailey-Kellogg, 2020). Predictions from the EpiPred webserver for the holdout set were performed, and precision and recall were computed (Table 5; Supplementary Table S4 and Fig. S1). EpiPred, which sets its epitope overlap cutoff at 30%, was unable to return predictions (EpiPred 3) for one of the smallest proteins. The predicted residues by AbAdapt were deemed epitope if their probabilities were above 0.5. AbAdapt achieved higher precision and recall than EpiPred, and the mean recall (0.662) was considerably higher than that of EpiPred (0.171). We also present representative epitope predictions for several holdout cases (Supplementary Fig. S4). As a reference, we also include native epitopes and predictions from the EpiPred webserver. There appear to be advantages to both EpiPred and AbAdapt. For example, for one of the worst AbAdapt case (4YDJ), there are elements of EpiPred’s first prediction, which captures some native epitope but also contains significant false-positive regions. At the other extreme, for one of AbAdapt’s better predictions (6II8), EpiPred’s top-three predictions appear to miss the epitope. In a typical case shown (4XAK), EpiPred’s first prediction overlapped with the native. One advantageous feature of EpiPred is its speed: results are returned in approximately 1 min, in comparison with AbAdapt’s multihour runtime.

**Table 5.** Comparison with EpiPred in holdout set (98)

	AbAdapt	EpiPred 1	EpiPred 2	EpiPred 3
Mean precision	0.160	0.098	0.107	0.102
Stdev precision	0.087	0.142	0.164	0.155
Mean recall	0.662	0.171	0.149	0.128
Stdev recall	0.267	0.249	0.226	0.200

The performance of AbAdapt epitope prediction was also evaluated using the PECAN benchmark (Pittala and Bailey-Kellogg, 2020), with the training and test set comprising 130 and 30 antibody–antigen crystal structure pairs, respectively, and the binding residue defined as any antigen (antibody) residue whose atom is within 4.5 Å of any antibody (antigen) atom. In order to compare with the published PECAN results, we reported the performance of Precision and Recall under a probability threshold of 0.5 and 0.379 the latter obtained maximizing Youden’s index (Youden, 1950), for five repetitions of the training process. There was a trade-off between precision and recall, as expected. Although the PR AUC values for PECAN were not reported, we estimated that antibody-specific AbAdapt AUCs (24%) were similar while antibody-agnostic AUCs (14%) were lower than those of PECAN, as summarized in Table 6.

**Table 6.** Comparison with PECAN antibody-specific epitope predictions

Prediction	Method	Precision Mean	Recall Mean	PR AUC Mean
AbAdapt	Antibody-specific	0.189	0.451	0.243
AbAdapt	Antibody-specific (0.379)	0.156	0.676	0.243
EpiPred		0.136	0.436	
PECAN	Conv2-Layer+Attn	0.154	0.672	<0.23
PECAN	Conv2-Layer+TL	0.157	0.730	<0.24
PECAN	Conv2-Layer+Attn+TL	0.158	0.628	<0.25

The AbAdapt paratope prediction performance was compared with the state-of-the-art predictor ProABC2 (Ambrosetti *et al.*, 2020b) and Parapred (Liberis *et al.*, 2018), as shown in Supplementary Table S5. The comparison was performed using two methods. We first evaluated the performance with our holdout set comprising 98 pairs by using their software (method 1). The statistics were calculated using the reported thresholds based on Youden’s index: 0.488 (Parapred) and 0.4 (ProABC-2); for AbAdapt, we used the calculated value of 0.395. Here, an improvement of AbAdapt paratope predictions over Parapred and ProABC-2 predictions could be observed. A second evaluation was performed with the Parapred benchmark (method 2) following the Parapred training procedure of 10 times 10-fold cross-validation. The set consisted of 277 crystal structures whose CDRs were defined according to the Chothia numbering scheme with two lateral residues at each end, and a paratope residue was defined as True if at least one atom was no greater than 4.5 Å from the antigen atom. The AbAdapt paratope predictions were binary labeled by maximizing Youden’s index, yielding thresholds of 0.439 for the Parapred benchmark. For this comparison, we show the prediction statistics for the thresholds 0.488 and 0.37 reported in Parapred and ProABC-2, respectively. AbAdapt performance for method 2 was lower than that of ProABC-2 or Parapred. These results indicate the sensitivity of all three methods to the training and testing methodology.

### 3.6 Webserver

The entire AbAdapt pipeline has been implemented as a webserver (https://sysimm.org/abadapt/). Example input and results pages are shown in Supplementary Figure S5. The epitope and paratope predictions are available for download as text files and can also be visualized as heatmaps in a molecular viewer. All inputs used in the LOOCV and holdout tests can be downloaded here as well.

## 4 Discussion

The development of AbAdapt was motivated, in part, by the recent emergence of various tools for repertoire-based analyses of immune responses. One can now obtain large numbers of paired antibody sequences from a routine blood sample, very often in situations where likely target antigens are known (i.e. infection or vaccination). One can, in addition, produce structural models of these antibodies in a high-throughput manner from such sequence data. Currently, available tools such as Lyra (Klausen *et al.*, 2015), AbodyBuilder (Leem *et al.*, 2016) and Repertoire Builder (Schritt *et al.*, 2019) all run on the seconds per model timescale, and more accurate high-throughput antibody modeling tools are likely on the way.

The emergence of high-throughput repertoire analysis technology presents a new challenge: to further extend antibody structures in order to characterize the target antigens and binding modes of antibodies of interest. Binding information could be used to understand the mechanism of action (e.g. neutralization activity) and to map out the landscape of binding modes for antibodies in disease cohorts with a well-defined and limited set of target antigens.

Here, we have attempted to build a working pipeline out of available tools, assess what worked and what did not, and suggest directions for future improvement. Our efforts were generally encouraging, as indicated by the query coverage and improvement in True Ranks across a large and diverse test set, along with the general agreement between LOOCV and holdout benchmarks. Although questions remain about the best balance between Piper and Hex poses, both the only-Piper and Piper–Hex AbAdapt pipelines produced better results than could be obtained by Piper alone. Also, the improvement in epitope prediction performance upon addition of antibody-specific features suggests a way of addressing this long-standing and important problem.

Areas where AbAdapt did not perform particularly well include: the quality of top-scoring poses and speed. The DockQ scores indicate that, by CAPRI standards, AbAdapt is not sampling very near-native complex models. The sensitivity of DockQ scores to antibody modeling errors indicates that this is likely a result of the combined effects of errors in the paratope residues of starting structures and the use of rigid docking. The most straightforward approach to addressing this issue is to use better antibody models. The release of highly accurate deep learning-based protein modeling methods (Baek *et al.*, 2021; Jumper *et al.*, 2021), along with the fact that AbAdapt can accept structures as well as sequences as input, makes this a realistic possibility. This is a direction we plan to explore in the near future. More recently, deep learning-based protein modeling methods have been applied successfully to multimer modeling, suggesting that protein docking may soon be subsumed by protein folding (Evans *et al.*, 2021). However, antibody–antigen docking still presents a significant challenge. In a recent benchmark of where AlphaFold and ClusPro were used in combination (Ghani *et al.*, 2021), the performance on antibody–antigen pairs was substantially lower than that for other targets. For example, for the best combination of methods (AlphaFold refined ClusPro plus AlphaFold models), no models deemed ‘Medium’ or ‘High’ by DockQ were generated by any of the tested methods, whereas for other targets, the frequency of ‘Medium’ models was similar to that of ‘High’ models. A speculative explanation for the AlphaFold/ClusPro results is that the methods used to generate multiple sequence alignment-based features in AlphaFold are not as appropriate for antibody–antigen pairs as they are for generic protein complexes (for which orthologous complexes exist in public databases, and a true interface signal can be seen in the MSA).

Although speed was not a primary focus of the current study, the fact that a typical antibody–antigen pair consumes multiple hours of CPU time (Supplementary Fig. S2), represents a limitation in the analysis of large numbers of sequences. To overcome this constraint, the wall-clock time was substantially reduced by using computational parallelism. However, if we assume that most high-throughput docking studies would be performed on a limited set of antigens using numerous antibodies, we can envision a scenario where a handful of representative antibodies are used to map ‘immunodominant’ epitope patches. Existing poses could then be recycled for local docking by new antibodies. We intend to explore this idea in the future. Taken together, AbAdapt represents a step toward integration of sequence and structural information that can be derived from antibody repertoires.

## Supplementary Material

vbac015_Supplementary_Data
