# Supplementary material for: AbAdapt: an adaptive approach to predicting antibody–antigen complex structures from sequence
Source: Bioinform Adv. 2022 Mar 7;2(1):vbac015. doi: 10.1093/bioadv/vbac015 (PMC9710585; doi:10.1093/bioadv/vbac015)
Supplement: vbac015_Supplementary_Data [file vbac015_supplementary_data.docx]

**AbAdapt: An adaptive approach to predicting antibody-antigen complex structures from sequence**

SUPPLEMENTARY MATERIALS

Ana Davila, Zichang Xu, Songling Li, John Rozewicki, Jan Wilamowski, Sergei Kotelnikov, Dima Kozakov, Shunsuke Teraguchi and Daron M. Standley

**S1**

Files detailing the query and template selection process can be downloaded directly from the AbAdapt server (https://gitlab.com/sysimm/abadapt-data). The files include:

1. Input files for LOOCV set
2. Input files for holdout set
3. Input files for ZDock set
4. Full-length RMSDs for each Ab and Ag chain, along with the overall sequence identity of each LOOCV antigen model to the structural template
5. Same RMSD and sequence identity data for holdout set

Folders for each query include:

1. Antibody PDB entry
2. Antigen PDB entry
3. Antibody-Antigen complex PDB entry
4. Native paratope
5. Native epitope
6. Antibody model used for docking
7. Antigen model used for docking
8. Reference complex combined antibody and antigen model superimposed on native complex)
9. Paratope and epitope RMSDs from the above superposition
10. Paratope and epitope residues numbered in terms of reference

**S2 Supplementary Figures**

**Figure S1. Precision and Recall for AbAdapt and Epipred.** The Precision (A) and Recall (B) are shown for AbAdapt interface residues predicted at 0.5 cutoff and top-three Epipred predictions, for 98 queries from the holdout set.

**Figure S2. Wall clock and CPU time dependence on antigen size.** The 100 holdout queries were run under conditions identical to those of the web server.


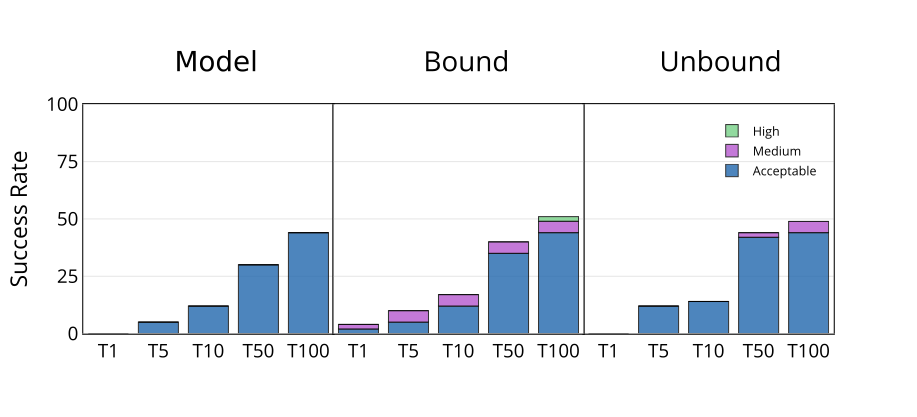


**Figure S3. DockQ Scores in ZDock benchmark.** The total number of Acceptable, Medium and High models for the 43 antibody-antigen pairs used in ZDock benchmark for the three conditions: homology models, bound structures and unbound structures.

**Figure S4. Comparison with Epipred.** Representative queries from the holdout set were submitted to the Epipred server. The antigen surfaces are colored by the Native epitope (row 1), top-three Epipred predictions (rows 2-4) and the AbAdapt epitope probability (row 6) and for 0.5 threshold (row 5). The AbAdapt ROC AUC, increasing from left to right, is indicated below row 6.


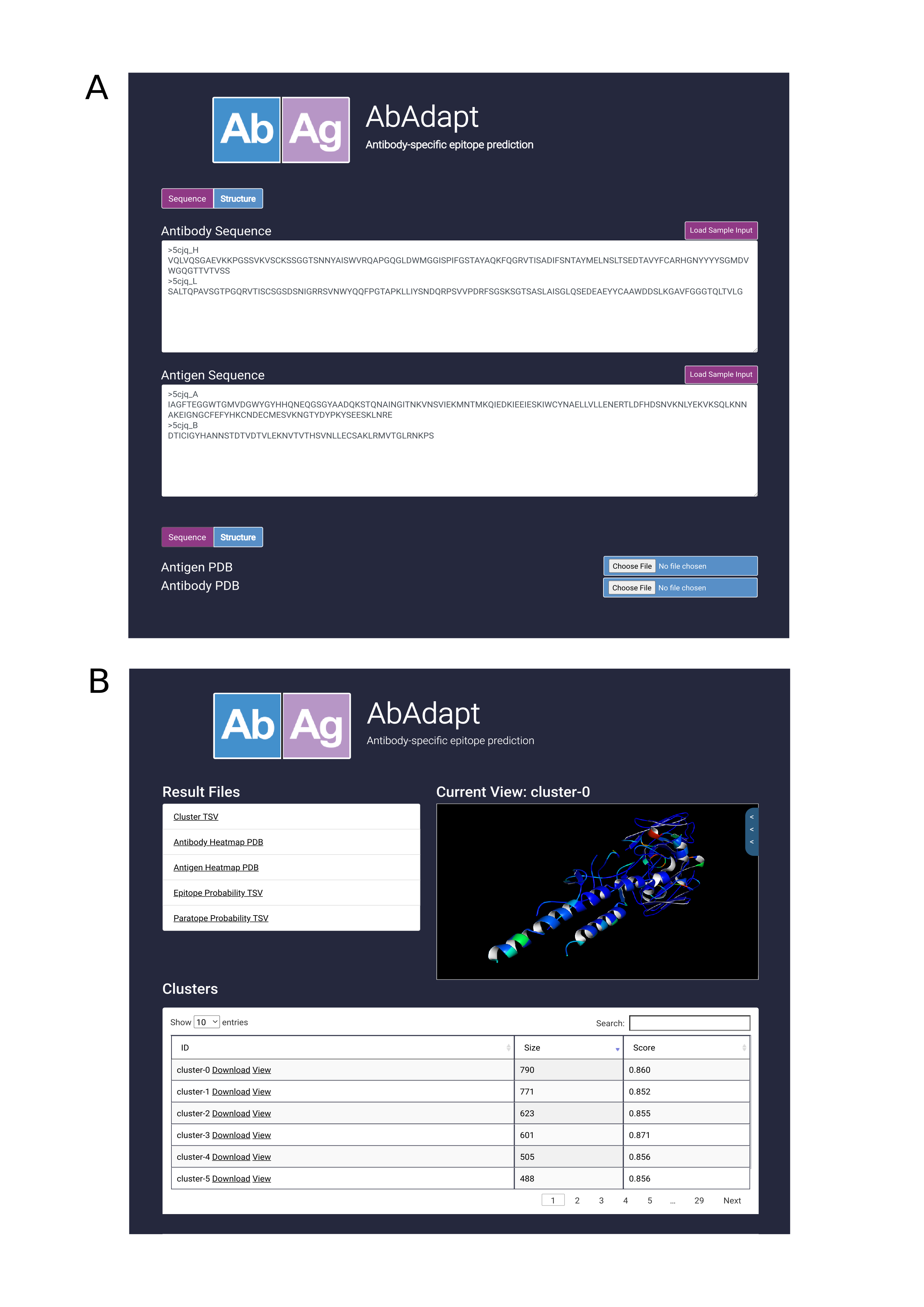
**Figure S5. AbAdapt webserver.** (**A**) The input consists of a paired (heavy-light chain) antibody sequence and an antigen sequence (multiple chains are allowed); if structures are available, they can be uploaded as well. (**B**) The output consists of a table of cluster representatives along with the cluster size and representative scores. Models can be viewed or downloaded.

**S3 Supplementary Tables**

**Table S1 Summary of ML models.** Summary of the six machine learning models used in AbAdapt.

| **Name** | **ML** | **Data** | **Features** | **Labels** |
| --- | --- | --- | --- | --- |
| **Initial_Paratope_ML** | DNN binary classifier | Antibody sequence and structure | amino acid (220), physico-chemical  properties (110), conservation (220), accessible  surface area (11) and surface shape (55) | 1 if binding; 0 otherwise |
| **Initial_Epitope_ML** | DNN binary classifier | Antigen sequence and structure | amino acid (220),  conservation (220), accessible  surface area (11) and surface shape (55) | Same as above |
| **Piper_Docking_ML** | Boosted Tree binary classifier | Piper docked poses | Ratios of  observed to expected contacts for each of the residue types in antibody (20), same ratios for antigen (20), number of clashes (1), joint-frequencies of  contacts/non-contacts in agreement/disagreement with paratope (3) and  epitope predictions (3). | 1 If IRMSD < 15 and fraction True Ab contacts > 0.5 and fraction True Ag contacts  > 0.5; 0 otherwise |
| **Hex_Docking_ML** | Boosted Tree binary classifier | Hex docked poses | Same as above | Same as above |
| **Piper_Hex_Docking_ML** | Boosted Tree regressor | Clusters of Piper and Hex poses | AbAdapt docking Score; number of (1), number of clashes (1), Hex or Piper docking energy (1); fraction of Piper poses in cluster (1), cluster size(1). | IRMSD^-2^ |
| **Final_Epitope_ML** | DNN binary classifier | Poses of Piper-Hex cluster representatives | Joint frequency of residue contacts and Piper-Hex Docking scores (9), conservation (220), accessible  surface area (11) and surface shape (55) | Same as for Initial_Epitope_ML |

**Table S2. Summary of DNN architectures.** Summary of the three neural networks architectures used in AbAdapt.

|  | **Initial_Paratope_ML** | **Initial_Epitope_ML** | **Final_Epitope_ML** |
| --- | --- | --- | --- |
| Feature size | 616 | 506 | 385 |
| Training set size | 56485 | 194048 | 194048 |
| Test set size | 9086 | 29007 | 29007 |
| Number of hidden layer | 7 | 11 | 7 |
| Total parameters | 2,580,225 | 2,460,481 | 2,624,257 |
| Trainable parameters | 2,572,929 | 2,451,265 | 2,616,769 |
| Non-trainable parameters | 7,296 | 9,216 | 7,488 |
| Activation Function | ReLU, Sigmoid | ReLU, Sigmoid | ReLU, Sigmoid |
| Dropout rates | 0.1 to 0.4 | 0.05 to 0.4 | 0.05 to 0.4 |
| Model Architecture | INPUT(616)  FC1(928)  Dropout(0.1)  FC2(64)  BatchNorm  Dropout(0.4)  FC3(736)  BatchNorm  Dropout(0.1)  FC4(512)  BatchNorm  Dropout(0.3)  FC5(960)  BatchNorm  Dropout(0.4)  FC6(576)  BatchNorm  Dropout(0.4)  FC7(800)  BatchNorm  Dropout(0.3)  FC8(1) | INPUT(506)  FC1(416)  Dropout(0.05)  FC2(544)  BatchNorm  Dropout(0.1)  FC3(800)  BatchNorm  Dropout(0.2)  FC4(608)  BatchNorm  Dropout(0.4)  FC5(640)  BatchNorm  Dropout(0.4)  FC6(160)  BatchNorm  Dropout(0.2)  FC7(128)  BatchNorm  Dropout(0.1)  FC8(800)  BatchNorm  Dropout(0.1)  FC9(480)  BatchNorm  Dropout(0.3)  FC10(96)  BatchNorm  Dropout(0.3)  FC11(352)  BatchNorm  Dropout(0.3)  FC12(1) | INPUT(385)  FC1(544)  Dropout(0.05)  FC2(480)  BatchNorm  Dropout(0.2)  FC3(640)  BatchNorm  Dropout(0.1)  FC4(544)  BatchNorm  Dropout(0.4)  FC5(768)  BatchNorm  Dropout(0.3)  FC6(896)  BatchNorm  Dropout(0.4)  FC7(416)  BatchNorm  Dropout(0.3)  FC8(1) |

**Table S3. Sensitivity to modeling inaccuracy.** Antigen models were binned according to their paratope RMSDs: 0-2A (Low), 2-4A (Med), 4-6 (High). Failure and Success (the ability to produce at least one True pose) or docking accuracy as classified by DockQ score was computed for each bin. The results are presented as 2x2 contingency tables.

**S3A. RMSD Low/Med vs Failed/Succeeded**

| **RMSD** | **Failed** | **Succeeded** | **SUM** |
| --- | --- | --- | --- |
| **Low** | 18 | 183 | 201 |
| **Med** | 34 | 298 | 332 |
| **SUM** | 52 | 481 | p=0.655 |

**S3B. RMSD Low/High vs Failed/Succeeded**

| **RMSD** | **Failed** | **Succeeded** | **SUM** |
| --- | --- | --- | --- |
| **Low** | 18 | 183 | 201 |
| **High** | 20 | 69 | 89 |
| **SUM** | 38 | 252 | **p=0.0025** |

**S3C. RMSD Med/High vs Failed/Succeeded**

| **RMSD** | **Failed** | **Succeeded** | **SUM** |
| --- | --- | --- | --- |
| **Med** | 34 | 298 | 332 |
| **High** | 20 | 69 | 89 |
| **SUM** | 54 | 367 | **p=0.0039** |

**S3D. RMSD Low/Med vs Incorrect/Acceptable (top 100)**

| **RMSD** | **Incorrect** | **Acceptable (t100)** | **SUM** |
| --- | --- | --- | --- |
| **Low** | 122 | 79 | 201 |
| **Med** | 235 | 235 | 332 |
| **SUM** | 357 | 176 | **p=0.018** |

**S3E. RMSD Low/High vs Incorrect/Acceptable (top 100)**

| **RMSD** | **Incorrect** | **Acceptable (t100)** | **SUM** |
| --- | --- | --- | --- |
| **low** | 122 | 79 | 201 |
| **high** | 73 | 16 | 89 |
| **SUM** | 195 | 95 | **p=0.00038** |

**S3F. RMSD Med/High vs Incorrect/Acceptable (top 100)**

| **RMSD** | **Incorrect** | **Acceptable (t100)** | **SUM** |
| --- | --- | --- | --- |
| **med** | 235 | 97 | 332 |
| **high** | 73 | 16 | 89 |
| **SUM** | 308 | 113 | **p=0.043** |

**S3G. RMSD Low/Med vs Incorrect/Acceptable (top 50)**

| **RMSD** | **Incorrect** | **Acceptable (t50)** | **SUM** |
| --- | --- | --- | --- |
| **low** | 139 | 62 | 201 |
| **med** | 269 | 63 | 332 |
| **SUM** | 408 | 125 | **p=0.0022** |

**S3H. RMSD Low/High vs Incorrect/Acceptable (top 50)**

| **RMSD** | **Incorrect** | **Acceptable (t50)** | **SUM** |
| --- | --- | --- | --- |
| **low** | 139 | 62 | 201 |
| **high** | 76 | 13 | 89 |
| **SUM** | 215 | 75 | **p=0.0035** |

**S3I. RMSD Med/High vs Incorrect/Acceptable (top 50)**

| **RMSD** | **Incorrect** | **Acceptable (t50)** | **SUM** |
| --- | --- | --- | --- |
| **med** | 269 | 63 | 332 |
| **high** | 76 | 13 | 89 |
| **SUM** | 345 | 76 | p=0.44 |

**S3J. RMSD Low/Med vs Incorrect/Acceptable (top 10)**

| **RMSD** | **Incorrect** | **Acceptable (t10)** | **SUM** |
| --- | --- | --- | --- |
| **low** | 181 | 20 | 201 |
| **med** | 306 | 26 | 332 |
| **SUM** | 487 | 46 | p=0.43 |

**S3K. RMSD Low/High vs Incorrect/Acceptable (top 10)**

| **RMSD** | **Incorrect** | **Acceptable (t10)** | **SUM** |
| --- | --- | --- | --- |
| **low** | 181 | 20 | 201 |
| **high** | 87 | 2 | 89 |
| **SUM** | 268 | 22 | **p=0.028** |

**S3L. RMSD Med/High vs Incorrect/Acceptable (top 10)**

| **RMSD** | **Incorrect** | **Acceptable (t10)** | **SUM** |
| --- | --- | --- | --- |
| **med** | 306 | 26 | 332 |
| **high** | 87 | 2 | 89 |
| **SUM** | 393 | 28 | **p=0.09** |

**Table S4. Comparison with Epipred.** The Precision and Recall were computed on the holdout set (98) for AbAdapt interface residues predicted at 0.5 cutoff and top-three Epipred predictions.

| **Query** | **Precision** | | | | **Recall** | | | | |
| --- | --- | --- | --- | --- | --- | --- | --- | --- | --- |
|  | Adapt | Epipred 1 | Epipred 2 | Epipred 3 | | Adapt | Epipred 1 | Epipred 2 | Epipred 3 |
| 1hysDCB | 0.031 | 0.000 | 0.000 | 0.000 | | 0.286 | 0.000 | 0.000 | 0.000 |
| 1kb5HLBA | 0.177 | 0.000 | 0.125 | 0.136 | | 0.759 | 0.000 | 0.103 | 0.103 |
| 1mlcBAE | 0.194 | 0.000 | 0.000 | 0.286 | | 0.737 | 0.000 | 0.000 | 0.316 |
| 1nsnHLS | 0.132 | 0.000 | 0.296 | 0.348 | | 0.375 | 0.000 | 0.333 | 0.333 |
| 1rvfHL1 | 0.095 | 0.000 | 0.029 | 0.290 | | 0.353 | 0.000 | 0.059 | 0.529 |
| 2adfHLA | 0.147 | 0.000 | 0.125 | 0.000 | | 0.682 | 0.000 | 0.136 | 0.000 |
| 2vxqHLA | 0.286 | 0.036 | 0.560 | 0.083 | | 0.667 | 0.048 | 0.667 | 0.048 |
| 3gjfHLA | 0.146 | 0.196 | 0.000 | 0.250 | | 0.905 | 0.429 | 0.000 | 0.333 |
| 3i50HLE | 0.230 | 0.000 | 0.379 | 0.000 | | 1.000 | 0.000 | 0.786 | 0.000 |
| 3idxHLG | 0.087 | 0.088 | 0.029 | 0.000 | | 0.476 | 0.143 | 0.048 | 0.000 |
| 3ldbCBA | 0.070 | 0.069 | 0.214 | 0.000 | | 0.381 | 0.095 | 0.286 | 0.000 |
| 3lqaHLG | 0.157 | 0.576 | 0.000 | 0.042 | | 0.810 | 0.905 | 0.000 | 0.048 |
| 3lzfHLA | 0.213 | 0.000 | 0.000 | 0.000 | | 0.680 | 0.000 | 0.000 | 0.000 |
| 3q1sHLI | 0.178 | 0.162 | 0.094 | 0.200 | | 0.813 | 0.375 | 0.188 | 0.250 |
| 4bz1HLA | 0.139 | 0.226 | 0.167 | 0.000 | | 0.769 | 0.538 | 0.308 | 0.000 |
| 4g3yHLC | 0.303 | 0.172 | 0.000 | 0.000 | | 0.769 | 0.192 | 0.000 | 0.000 |
| 4hf5HLA | 0.291 | 0.000 | 0.231 | 0.346 | | 0.962 | 0.000 | 0.231 | 0.346 |
| 4hfuHLA | 0.315 | 0.129 | 0.000 | 0.000 | | 0.793 | 0.138 | 0.000 | 0.000 |
| 4irzHLA | 0.178 | 0.000 | 0.000 | 0.069 | | 0.692 | 0.000 | 0.000 | 0.077 |
| 4janHLG | 0.298 | 0.000 | 0.031 | 0.222 | | 0.708 | 0.000 | 0.042 | 0.250 |
| 4k24HLU | 0.119 | 0.000 | 0.103 | 0.000 | | 1.000 | 0.000 | 0.267 | 0.000 |
| 4lvoCBA | 0.085 | 0.438 | 0.000 | 0.000 | | 0.789 | 0.737 | 0.000 | 0.000 |
| 4m7lHLT | 0.180 | 0.313 | 0.067 | 0.000 | | 0.643 | 0.357 | 0.071 | 0.000 |
| 4od2BAS | 0.175 | 0.333 | 0.259 | 0.038 | | 0.526 | 0.526 | 0.368 | 0.053 |
| 4pd4JKE | 0.250 | 0.250 | 0.136 | 0.000 | | 0.889 | 0.444 | 0.167 | 0.000 |
| 4q6iHLC | 0.140 | 0.000 | 0.000 | 0.250 | | 1.000 | 0.000 | 0.000 | 0.429 |
| 4xakDEB | 0.246 | 0.368 | 0.000 | 0.120 | | 0.778 | 0.778 | 0.000 | 0.167 |
| 4ydjABI | 0.222 | 0.265 | 0.000 | 0.333 | | 0.207 | 0.310 | 0.000 | 0.310 |
| 4ydlHLG | 0.187 | 0.000 | 0.000 | 0.240 | | 0.903 | 0.000 | 0.000 | 0.194 |
| 5d1xBAE | 0.211 | 0.333 | 0.103 | 0.000 | | 0.833 | 0.556 | 0.167 | 0.000 |
| 5d1zDCI | 0.140 | 0.024 | 0.211 | 0.300 | | 0.765 | 0.059 | 0.471 | 0.529 |
| 5fuuMNB | 0.074 | 0.108 | 0.000 | 0.162 | | 0.857 | 0.571 | 0.000 | 0.857 |
| 5hbtDCB | 0.223 | 0.000 | 0.000 | 0.407 | | 1.000 | 0.000 | 0.000 | 0.524 |
| 5kteHLA | 0.189 | 0.212 | 0.000 | 0.000 | | 0.500 | 0.500 | 0.000 | 0.000 |
| 5w08OPE | 0.208 | 0.000 | 0.000 | 0.269 | | 0.667 | 0.000 | 0.000 | 0.292 |
| 5w3oDEBAC | 0.098 | 0.000 | 0.000 | 0.133 | | 0.462 | 0.000 | 0.000 | 0.154 |
| 5w4lFIA | 0.144 | 0.000 | 0.033 | 0.037 | | 0.815 | 0.000 | 0.037 | 0.037 |
| 5x2pJKC | 0.000 | 0.000 | 0.000 | 0.000 | | 0.000 | 0.000 | 0.000 | 0.000 |
| 5xbmBAC | 0.085 | 0.000 | 0.000 | 0.000 | | 0.526 | 0.000 | 0.000 | 0.000 |
| 5y11ABC | 0.070 | 0.179 | 0.077 | 0.000 | | 0.421 | 0.263 | 0.105 | 0.000 |
| 5zs0BAC | 0.118 | 0.000 | 0.000 | 0.414 | | 0.533 | 0.000 | 0.000 | 0.800 |
| 6a4kKOD | 0.111 | 0.000 | 0.000 | 0.000 | | 0.867 | 0.000 | 0.000 | 0.000 |
| 6apbHLC | 0.000 | 0.457 | 0.000 | 0.000 | | 0.000 | 0.800 | 0.000 | 0.000 |
| 6aq7HLA | 0.298 | 0.353 | 0.036 | 0.118 | | 0.737 | 0.632 | 0.053 | 0.105 |
| 6azzCBA | 0.233 | 0.000 | 0.438 | 0.107 | | 0.955 | 0.000 | 0.636 | 0.136 |
| 6bp2HLA | 0.296 | 0.107 | 0.556 | 0.000 | | 0.615 | 0.115 | 0.577 | 0.000 |
| 6bpaEFD | 0.014 | 0.000 | 0.000 | 0.000 | | 0.059 | 0.000 | 0.000 | 0.000 |
| 6dfjHLE | 0.232 | 0.000 | 0.037 | 0.421 | | 0.684 | 0.000 | 0.053 | 0.421 |
| 6didKEG | 0.045 | 0.156 | 0.032 | 0.000 | | 0.136 | 0.227 | 0.045 | 0.000 |
| 6eayHLAB | 0.153 | 0.135 | 0.000 | 0.481 | | 0.406 | 0.156 | 0.000 | 0.406 |
| 6h2yHLD | 0.167 | 0.314 | 0.346 | 0.000 | | 0.690 | 0.379 | 0.310 | 0.000 |
| 6higHLB | 0.156 | 0.321 | 0.042 | 0.667 | | 0.556 | 0.500 | 0.056 | 0.333 |
| 6ii8JKE | 0.413 | 0.000 | 0.000 | 0.000 | | 0.792 | 0.000 | 0.000 | 0.000 |
| 6j14ABG | 0.229 | 0.000 | 0.476 | 0.000 | | 0.917 | 0.000 | 0.833 | 0.000 |
| 6k7oBCP | 0.231 | 0.000 | 0.200 | 0.000 | | 1.000 | 0.000 | 0.267 | 0.000 |
| 6m3bCBA | 0.211 | 0.412 | 0.100 | 0.000 | | 0.964 | 0.500 | 0.107 | 0.000 |
| 6mfpHLG | 0.167 | 0.000 | 0.152 | 0.000 | | 0.957 | 0.000 | 0.217 | 0.000 |
| 6midHLE | 0.294 | 0.000 | 0.000 | 0.000 | | 0.926 | 0.000 | 0.000 | 0.000 |
| 6n5eEDA | 0.191 | 0.000 | 0.500 | 0.125 | | 0.739 | 0.000 | 0.522 | 0.130 |
| 6n8dFEC | 0.080 | 0.000 | 0.000 | 0.000 | | 0.435 | 0.000 | 0.000 | 0.000 |
| 6nc2HLBA | 0.030 | 0.024 | 0.000 | 0.000 | | 0.500 | 0.167 | 0.000 | 0.000 |
| 6nmtBAC | 0.159 | 0.030 | 0.111 | 0.190 | | 0.778 | 0.056 | 0.167 | 0.222 |
| 6p67ABK | 0.127 | 0.000 | 0.233 | 0.545 | | 0.619 | 0.000 | 0.333 | 0.571 |
| 6p95DEa | 0.211 | 0.244 | 0.000 | 0.000 | | 0.952 | 0.476 | 0.000 | 0.000 |
| 6plkIMF | 0.118 | 0.000 | 0.160 | 0.571 | | 0.125 | 0.000 | 0.250 | 0.750 |
| 6ppgHLFG | 0.143 | 0.000 | 0.000 | 0.214 | | 0.773 | 0.000 | 0.000 | 0.273 |
| 6qd7HLD | 0.313 | 0.033 | 0.438 | 0.000 | | 0.882 | 0.059 | 0.824 | 0.000 |
| 6qd8QYB | 0.044 | 0.000 | 0.000 | 0.190 | | 0.167 | 0.000 | 0.000 | 0.333 |
| 6qnoHLB | 0.022 | 0.000 | 0.000 | 0.238 | | 0.200 | 0.000 | 0.000 | 0.500 |
| 6u38HLB | 0.075 | 0.000 | 0.000 | 0.000 | | 0.867 | 0.000 | 0.000 | 0.000 |
| 6urmDEC | 0.241 | 0.000 | 0.481 | 0.000 | | 0.875 | 0.000 | 0.542 | 0.000 |
| 6utkHLG | 0.093 | 0.000 | 0.000 | 0.000 | | 1.000 | 0.000 | 0.000 | 0.000 |
| 6uymABF | 0.246 | 0.172 | 0.036 | 0.000 | | 1.000 | 0.313 | 0.063 | 0.000 |
| 6vgrHLA | 0.067 | 0.000 | 0.000 | 0.000 | | 1.000 | 0.000 | 0.000 | 0.000 |
| 6vx4HKFG | 0.092 | 0.000 | 0.000 | 0.133 | | 0.609 | 0.000 | 0.000 | 0.174 |
| 6w52HLA | 0.164 | 0.407 | 0.000 | 0.077 | | 0.818 | 1.000 | 0.000 | 0.091 |
| 6wdtHLCB | 0.098 | 0.000 | 0.000 | 0.000 | | 1.000 | 0.000 | 0.000 | 0.000 |
| 6werEFB | 0.070 | 0.000 | 0.000 | 0.000 | | 0.409 | 0.000 | 0.000 | 0.000 |
| 6wmwHLB | 0.044 | 0.114 | 0.480 | 0.000 | | 0.211 | 0.211 | 0.632 | 0.000 |
| 6wqoEFD | 0.053 | 0.179 | 0.000 | 0.000 | | 0.353 | 0.412 | 0.000 | 0.000 |
| 6xgcIJF | 0.200 | 0.255 | 0.152 | 0.000 | | 1.000 | 0.667 | 0.278 | 0.000 |
| 6xlqEFD | 0.193 | 0.000 | 0.000 | 0.037 | | 0.842 | 0.000 | 0.000 | 0.053 |
| 6xrtHLE | 0.060 | 0.000 | 0.000 | 0.000 | | 0.833 | 0.000 | 0.000 | 0.000 |
| 6y9aHLB | 0.128 | 0.000 | 0.000 | 0.000 | | 1.000 | 0.000 | 0.000 | 0.000 |
| 7bq5CDA | 0.068 | 0.000 | 0.000 | 0.069 | | 0.714 | 0.000 | 0.000 | 0.286 |
| 7ce2ZBA | 0.063 | 0.063 | 0.000 | 0.400 | | 0.364 | 0.091 | 0.000 | 0.455 |
| 7cgwABC | 0.293 | 0.355 | 0.300 | 0.000 | | 0.857 | 0.393 | 0.321 | 0.000 |
| 7dnhHLAE | 0.164 | 0.000 | 0.000 | 0.000 | | 0.563 | 0.000 | 0.000 | 0.000 |
| 7jtgABE | 0.149 | 0.281 | 0.357 | 0.043 | | 0.556 | 0.500 | 0.556 | 0.056 |
| 7jv4DEB | 0.115 | 0.000 | 0.086 | 0.000 | | 0.300 | 0.000 | 0.300 | 0.000 |
| 7kcrHLA | 0.100 | 0.000 | 0.000 | 0.000 | | 0.500 | 0.000 | 0.000 | 0.000 |
| 7kf9ILC | 0.236 | 0.000 | 0.552 | 0.000 | | 0.875 | 0.000 | 0.667 | 0.000 |
| 7kr5OPE | 0.318 | 0.043 | 0.000 | 0.000 | | 0.778 | 0.222 | 0.000 | 0.000 |
| 7kylXYZ | 0.186 | 0.103 | 0.360 | 0.308 | | 0.611 | 0.167 | 0.500 | 0.222 |
| 7labYXB | 0.019 | 0.000 | 0.000 | 0.000 | | 0.087 | 0.000 | 0.000 | 0.000 |
| 7m7wABS | 0.182 | 0.400 | 0.000 | 0.000 | | 0.783 | 0.522 | 0.000 | 0.000 |
| 7m7wCDS | 0.099 | 0.125 | 0.520 | 0.040 | | 0.381 | 0.190 | 0.619 | 0.048 |
| 7nd4HLC | 0.345 | 0.000 | 0.000 | 0.000 | | 0.500 | 0.000 | 0.000 | 0.000 |

**Table S5. Comparison with ProABC-2 and Parapred Paratope Predictions.**

| **Method** | **Predictor** | **TEST set** | **Threshold** | **ROC AUC** | **MCC** | **F1 Score** |
| --- | --- | --- | --- | --- | --- | --- |
| 1 | AbAdapt | AbAdapt | 0.395 | 0.891 | 0.552 | 0.644 |
| 1 | Parapred | AbAdapt | 0.488 | 0.702 | 0.250 | 0.445 |
| 1 | ProABC-2 | AbAdapt | 0.400 | 0.861 | 0.469 | 0.575 |
| 2 | AbAdapt | Parapred | 0.439 | 0.868 | 0.536 | 0.683 |
| 2 | Parapred | Parapred | 0.488 | 0.878 | 0.554 | 0.690 |
| 2 | ProABC-2 | Parapred | 0.370 | 0.910 | 0.560 | 0.620 |
